# Supplementary material for: Structural basis for uracil removal from DNA by human SMUG1
Source: Nat Commun. 2026 Jun 2;17:4809. doi: 10.1038/s41467-026-72937-0 (PMC13230825; doi:10.1038/s41467-026-72937-0)
Supplement: Supplementary file 1 — Supplementary Information [file 41467_2026_72937_MOESM1_ESM.pdf]

Supplementary Information

Structural basis for uracil removal from DNA by human SMUG1

Julian M. Ludäscher[1], Emma Scaletti Hutchinson[1], Guillem Vila-Julià[2], Ann-Sofie Jemth[3], Saher Shahid[1], Elisee Wiita[3], Israel Cabeza de Vaca[2], Szymon Pach[2], Lukas Gajdos[4], Swati Aggarwal[5,6], Ellen Walse[1], Oliver Mortusewicz[3], Thomas Helleday[3,] Jens Carlsson[2], Pål Stenmark[1,*]

[1]Department of Biochemistry and Biophysics, Stockholm University, SE-10691 Stockholm, Sweden

[2]Science for Life Laboratory, Department of Cell and Molecular Biology, Uppsala University, BMC, Box 596, SE-75124 Uppsala, Sweden

[3]Science for Life Laboratory, Department of Oncology-Pathology, Karolinska Institute, SE-17177 Stockholm, Sweden

[4]Large Scale Structures group, Institut Laue-Langevin (ILL), 38000 Grenoble, France

[5]European Spallation Source (ESS), SE-22484 Lund, Sweden

[6]Division of Computational Chemistry, Lund University, SE-22262 Lund, Sweden

*To whom correspondence should be addressed: stenmark@dbb.se

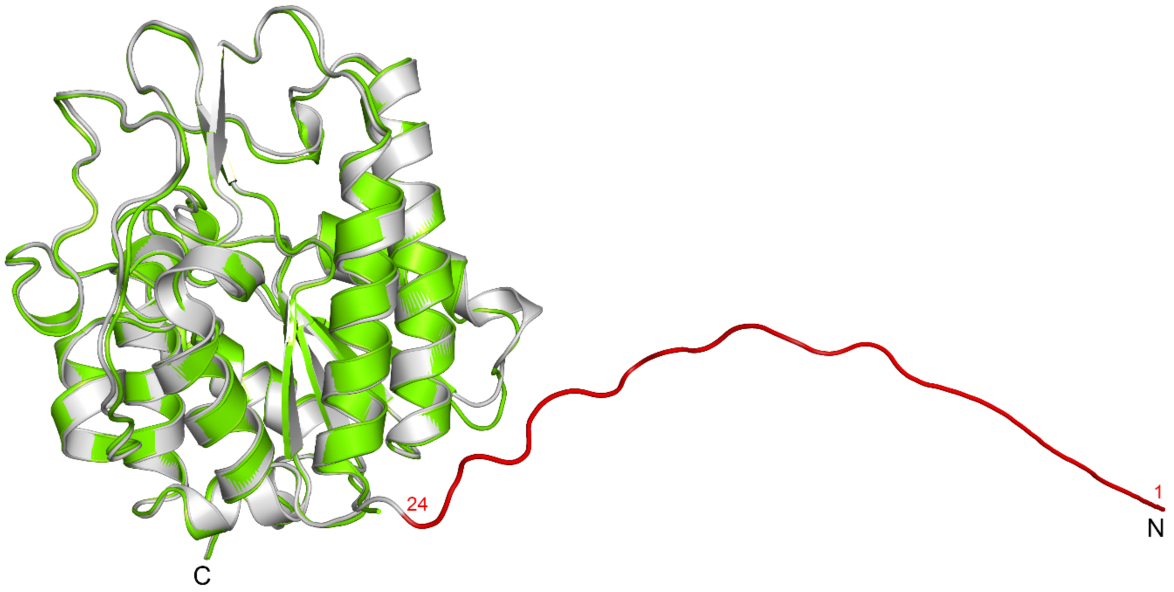

Supplementary Figure 1: Comparison of hSMUG1 X-ray crystal structure with AlphaFold model. An AlphaFold model was produced using the AlphaFold webserver (https://alphafold.ebi.ac.uk) and the amino acid sequence of hSMUG1 (residues 1-270, UniProt: Q53HV7) as the input. Individual monomers are shown as cartoon representations coloured green (hSMUG1 X-ray structure) or light grey (Alphafold model). Cα-atom superposition of the two structures is associated with an RMSD of 0.56 Å. Residues 1-24 shown in the hSMUG1 AlphaFold model are coloured red. Figure produced with PyMOL (version 3.0.4, Schrödinger).

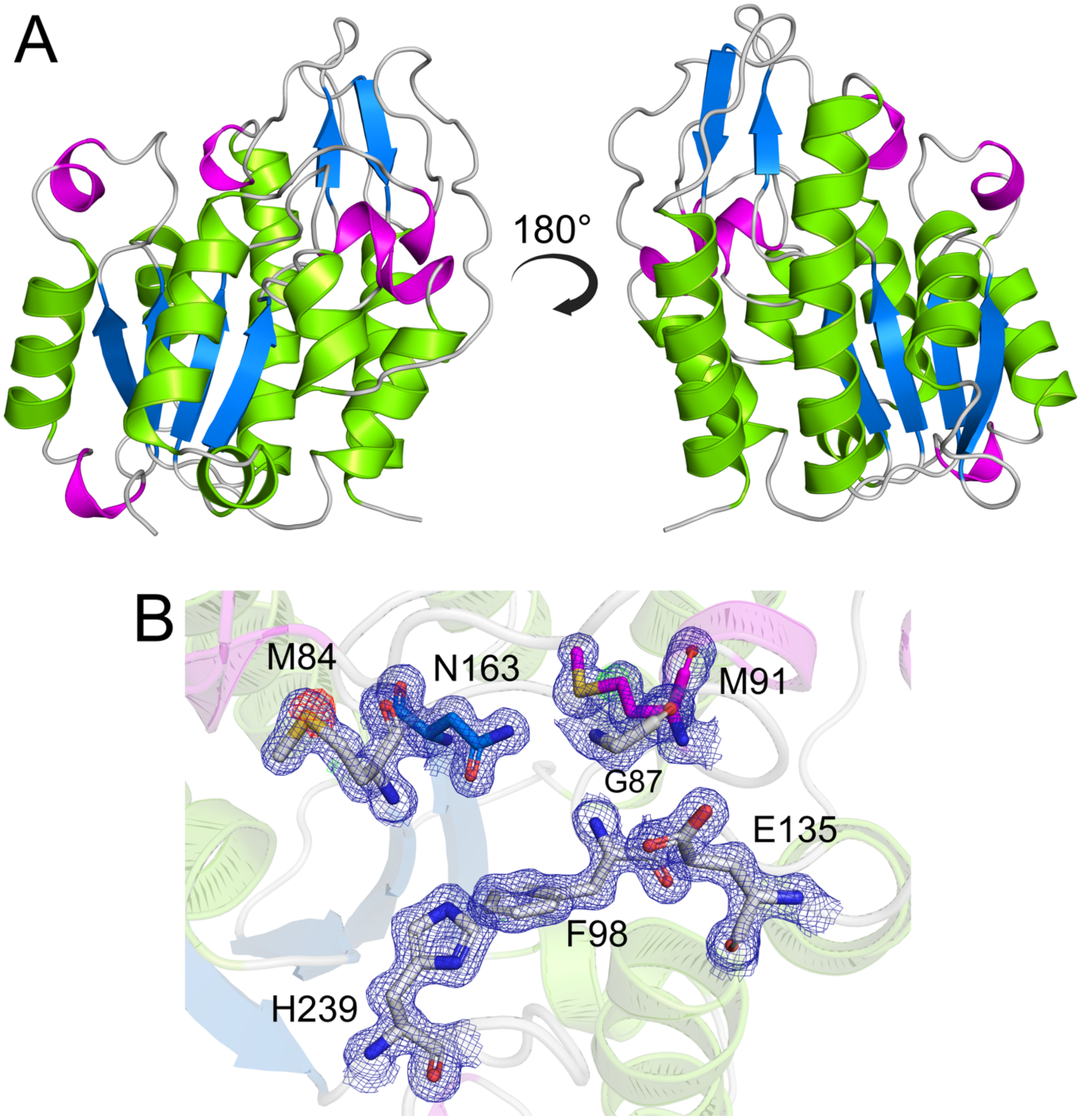

Supplementary Figure 2: X-ray crystal structure of apo hSMUG1. (A) Cartoon representation of the hSMUG1 monomer, coloured according to secondary structure. Alpha-helices are coloured green, beta-strands are coloured blue, 3_{10}-helices are coloured magenta and loop regions are coloured light grey. **(B)** Electron density quality for the apo hSMUG1 uracil binding pocket. Amino acids are depicted as sticks; C atoms are coloured according to secondary structure shown in **A**, O atoms red, N atoms dark blue and S atoms gold. The $2F_o$-F_c electron density map (blue) for residues in the uracil binding pocket is contoured at 1.8σ and the F_o-F_c electron density maps are contoured at $+4.0\sigma$ (green) and -4.0σ (red). Figure produced with PyMOL (version 3.0.4, Schrödinger).

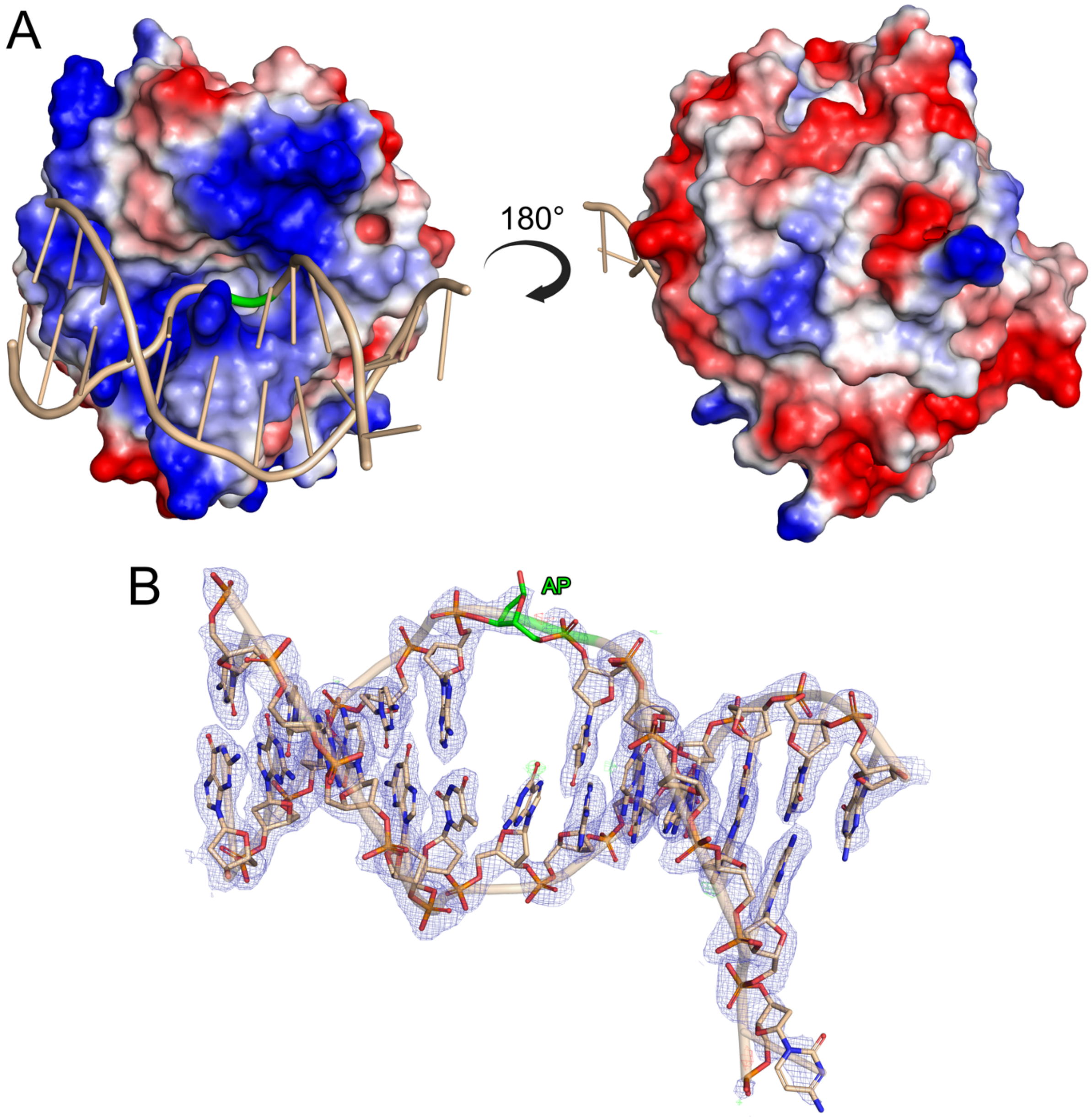

Supplementary Figure 3: hSMUG1 in complex with dsDNA. (A) The electrostatic potential of hSMUG1 was calculated with APBS[97] in the range −5 kT (red, negative potential) to +5 kT (blue, positive potential). The dsDNA substrate is shown as a beige cartoon representation. The AP site in the dsDNA substrate is coloured green. **(B)** Electron density for the dsDNA product. Deoxynucleotides are shown as sticks; C atoms are coloured beige, N atoms dark blue, O atoms red and P atoms orange. The $2F_o$-F_c electron density map (blue) is contoured at 1.0σ and the F_o-F_c electron density maps are contoured at $+3.0\sigma$ (green) and -3.0σ (red). Figure produced with PyMOL (version 3.0.4, Schrödinger).

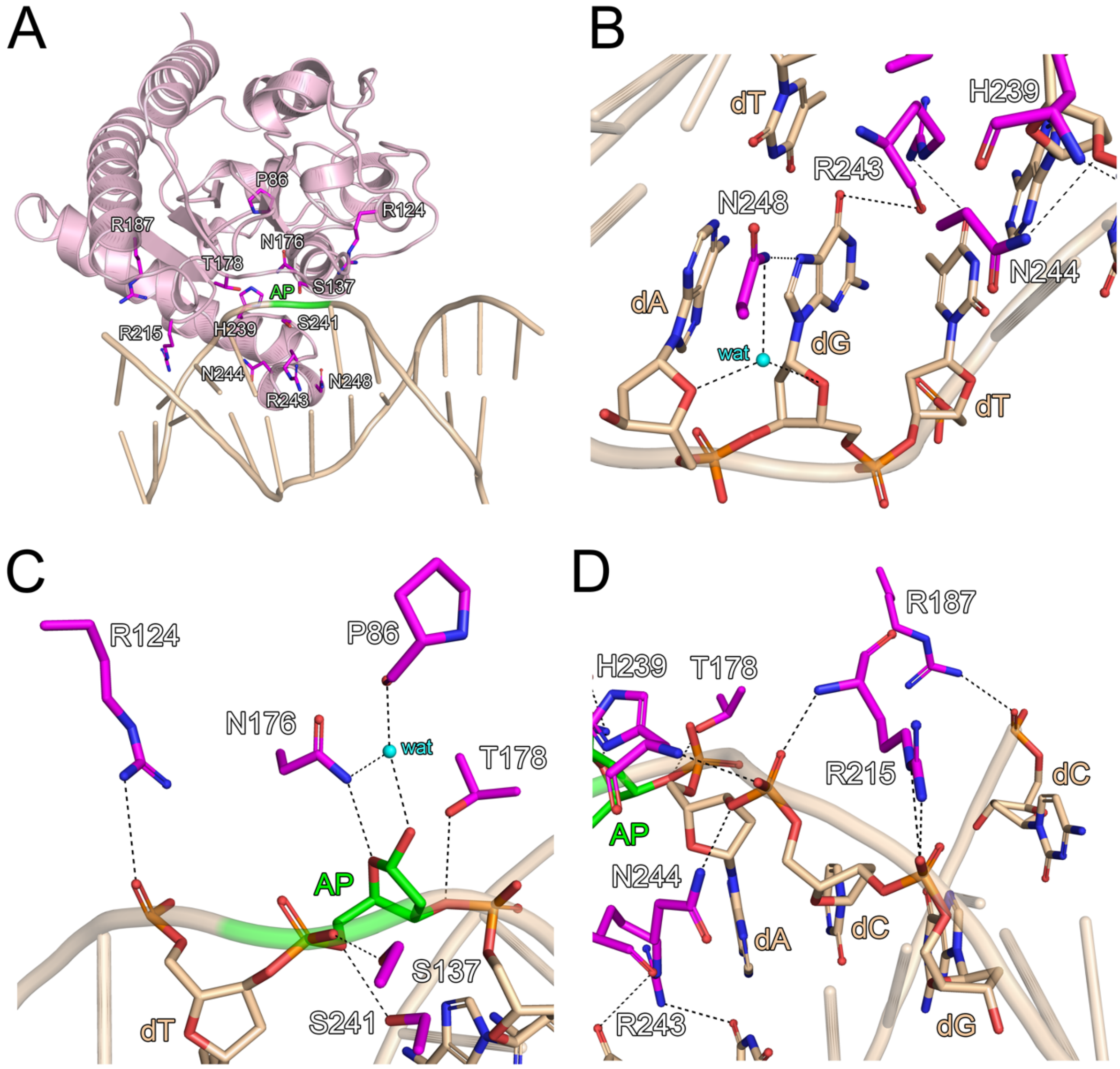

Supplementary Figure 4: H-bond interactions observed in hSMUG1-dsDNA complex. (A) Cartoon representation of hSMUG1 monomer (light pink) with dsDNA (beige). The AP site in the dsDNA substrate is coloured green. Amino acids which interact with dsDNA though either direct or water mediated hydrogen bonds are shown as sticks. **(B-D)** H-bond interactions for DNA-interacting residues. Hydrogen bonds are shown as dashed lines. Deoxyguanosine (dG), deoxyadenosine (dA), deoxycytosine (dC) and deoxythymidine (dT) are shown as sticks. C atoms are coloured magenta (hSMUG1), green (AP site of dsDNA) or beige (relevant deoxynucleotides), O atoms red, N atoms dark blue and P atoms orange. Water molecules are depicted as cyan spheres. Figure produced with PyMOL (version 3.0.4, Schrödinger).

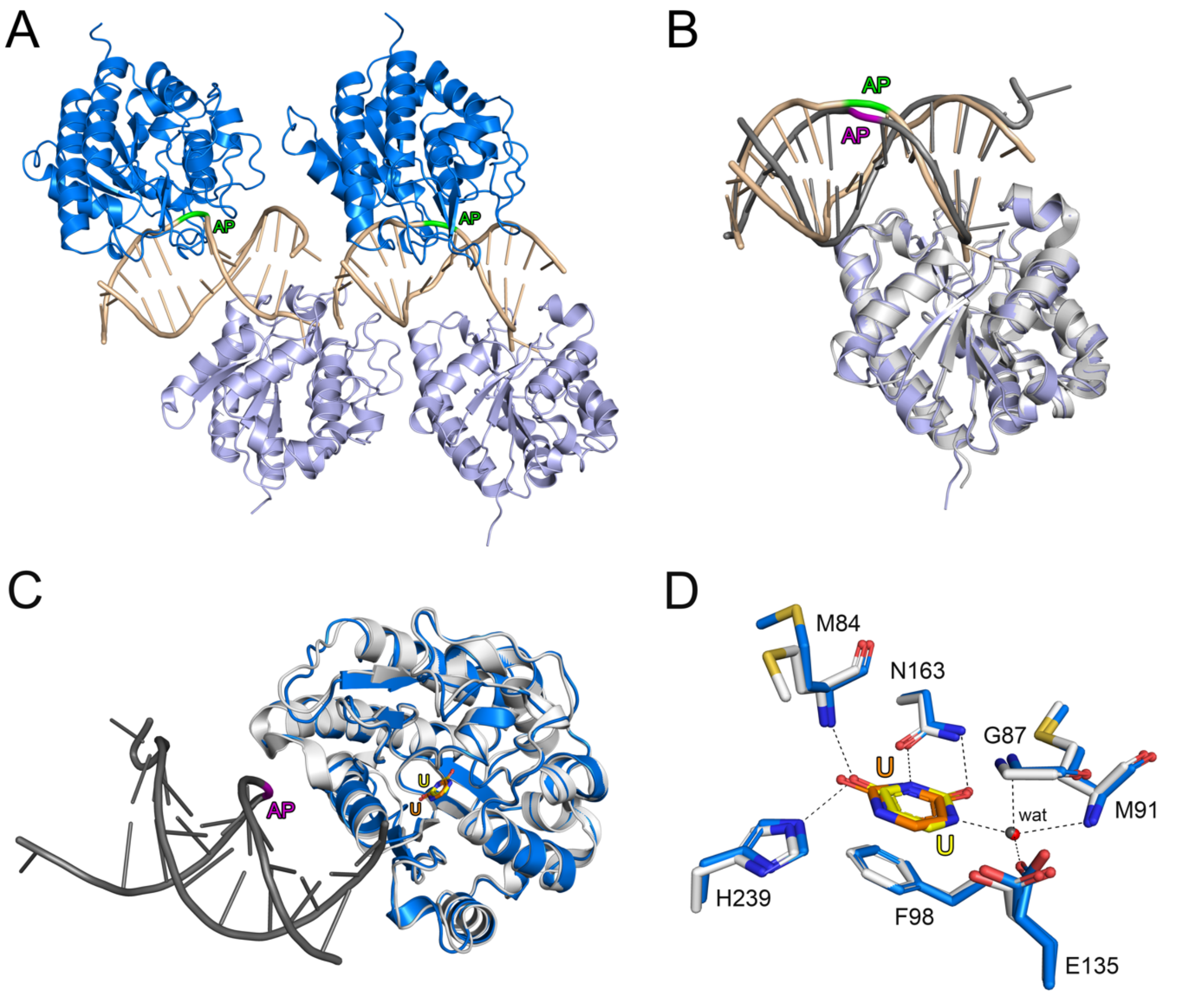

Supplementary Figure 5: Comparison of human and frog SMUG1 X-ray crystal structures. (A) Cartoon representation of hSMUG1 monomers from the asymmetric unit (blue) and hSMUG1 monomers related by crystallographic symmetry (light blue). The dsDNA substrate is shown as a beige cartoon representation. The AP site in the dsDNA substrate is coloured green. Blue monomers indicate a dsDNA-product binding mode, whereas light blue monomers show a non-productive DNA "end-binding" mode. **(B)** Comparison of hSMUG1 in dsDNA end-binding mode with *Xenopus laevis* SMUG1 (xSMUG1, PDB ID: 1OE4) where the protein monomer and dsDNA are coloured light grey and dark grey, respectively. In the xSMUG1 structure the AP site in the dsDNA is coloured magenta. Cα-atom superposition of the two structures is associated with an RMSD of 0.71 Å. **(C)** Comparison of hSMUG1-uracil (blue) with xSMUG1 (light grey) bound to both dsDNA (dark grey) and uracil (PDB ID: 1OE5). Uracil (U) is shown as sticks. C atoms are coloured yellow (hSMUG1-uracil) or orange (xSMUG1-dsDNA-uracil), O atoms red and N atoms dark blue. Cα-atom superposition of the two structures is associated with an RMSD of 0.63 Å. **(D)** Zoomed-in view of uracil binding pocket for structures presented in panel **C**. Amino acid side chains are depicted as sticks. Amino acid numbering corresponds to the hSMUG1 structure, where H-bonds are shown as black dashed lines. Water molecules are depicted as red (hSMUG1-uracil) or dark grey (xSMUG1-dsDNA-uracil) spheres. Figure produced with PyMOL (version 3.0.4, Schrödinger).

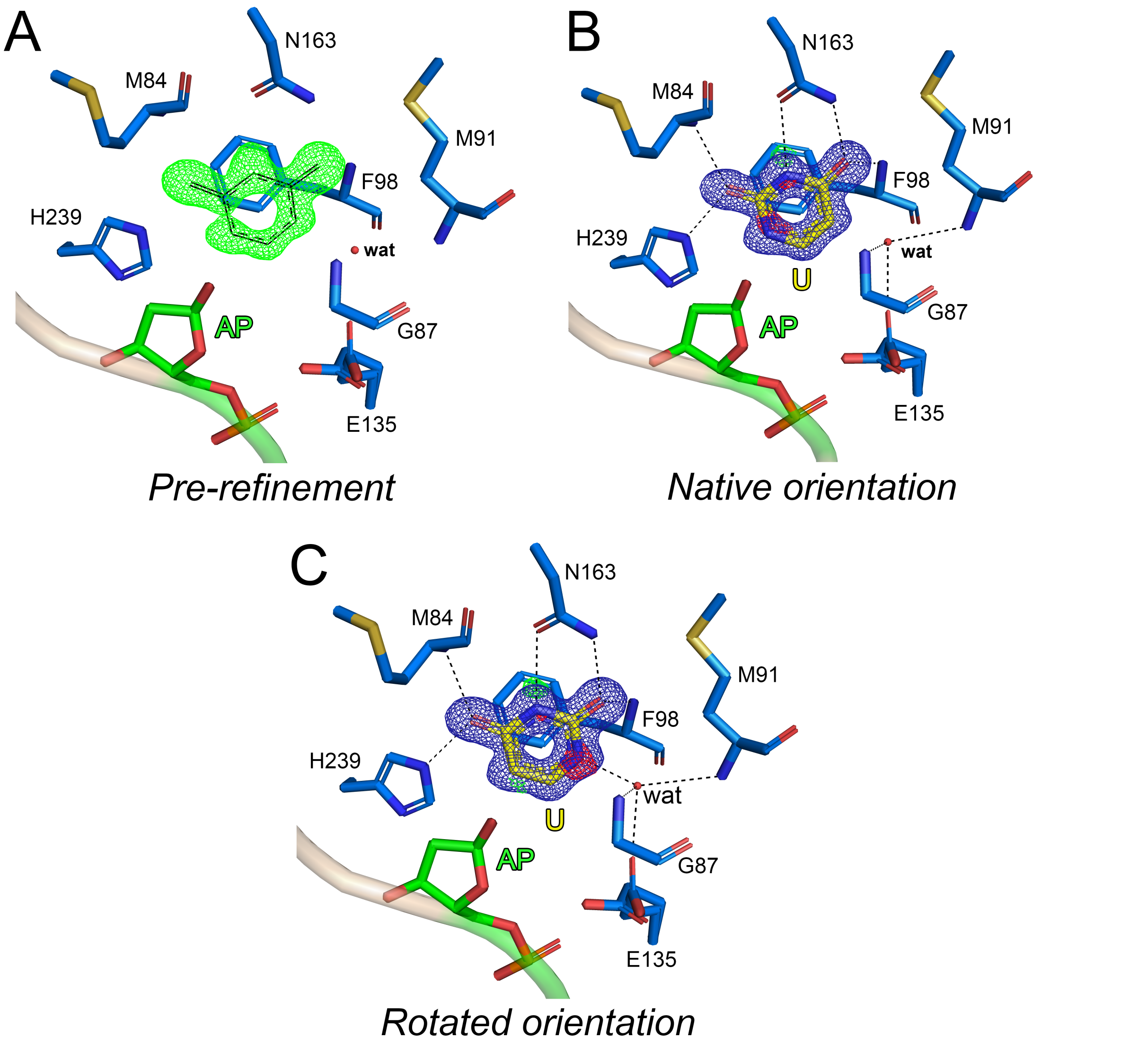

Supplementary Figure 6: hSMUG1 uracil-binding modes. hSMUG1-uracil (blue) was superimposed with hSMUG1-dsDNA (only dsDNA is shown). Uracil (U) is shown as sticks. C atoms are coloured yellow (U), O atoms red and N atoms dark blue. The dsDNA substrate is shown as a beige cartoon representation. The AP site in the dsDNA substrate is depicted as green sticks. **(A)** F_o-F_c omit map (green, 3.0σ) prior to uracil addition and refinement. **(B)** Refinement of uracil in native orientation **(C)** Refinement of uracil in the rotated orientation. In panels **B** and **C**, the 2F_o-F_c electron density maps around uracil are contoured at 1.8σ and the F_o-F_c electron density maps are contoured at +4.0σ (green) and -4.0σ (red). Figure produced with PyMOL (version 3.0.4, Schrödinger).

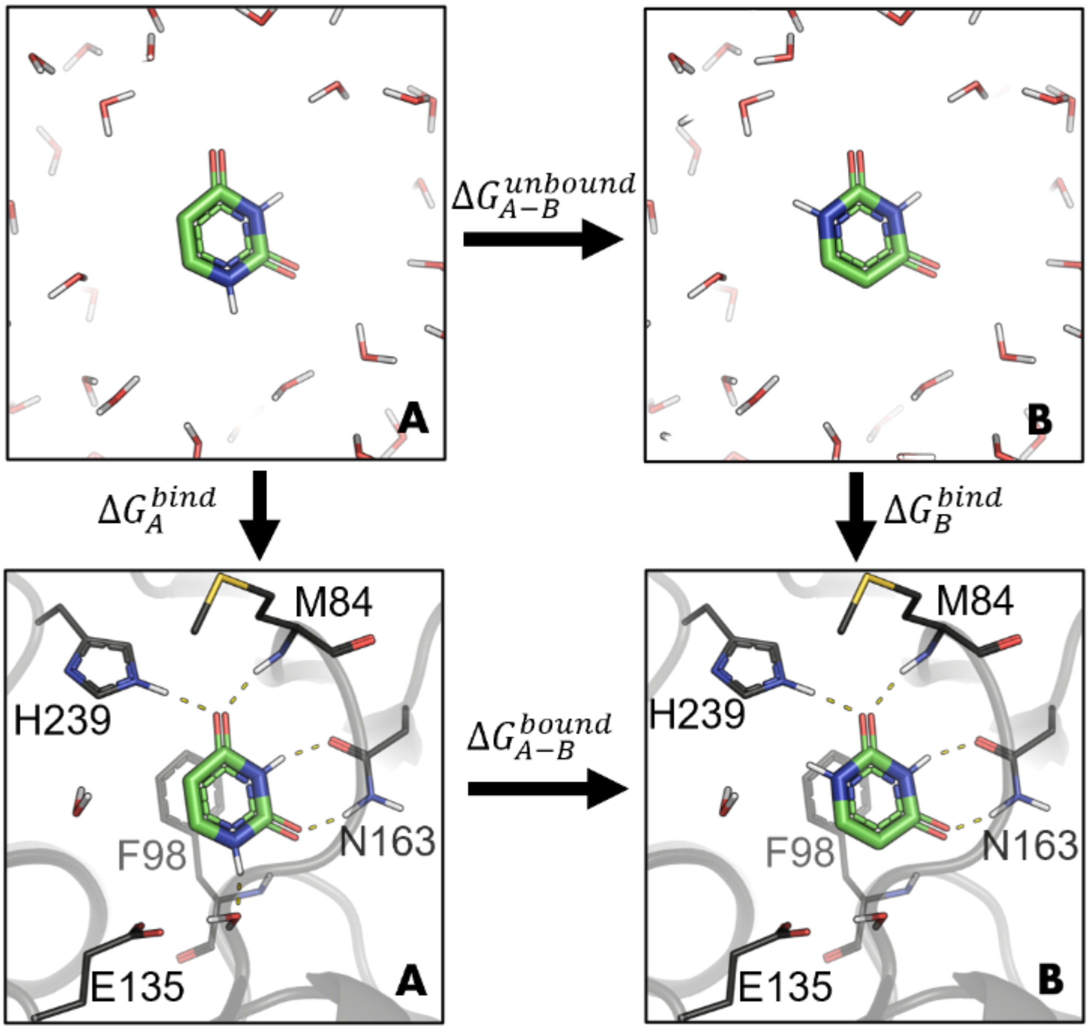

Supplementary Figure 7. Thermodynamic cycle for calculating the relative binding free energy (ΔΔG) between two alternative uracil-binding modes. The relative binding free energy between the rotated **(A)** and native poses **(B)**, $\Delta\Delta G = \Delta G_{B}^{bind} - \Delta G_{A}^{bind}$, can be calculated as the difference between the alchemical transformations in the unbound ($\Delta G_{A-B}^{unbound}$) and bound (ΔG_{A-B}^{bound}) states. hSMUG1 is shown as a grey cartoon. Uracil is represented as green sticks whereas key residues and water molecules are represented as lines. Carbon atoms are coloured green, oxygen red, nitrogen dark blue and sulphur gold.

Supplementary Table 1. Free energy differences between rotated and native uracil binding poses (in kcal/mol) for each MD simulation replica in the unbound ($\Delta G_{A-B}^{unbound}$) and bound (ΔG_{A-B}^{bound}) states. Forward and backward transformations are reported separately for both the unbound and bound states alongside their respective standard errors of the mean (SEM). The last two rows show the average values and the associated SEM.

Replica	$\Delta G_{A-B}^{unbound*}$		ΔG_{A-B}^{bound}	
	Forward	Backward	Forward	Backward
1	-0.13 ± 0.14	-0.04 ± 0.11	2.42 ± 0.36	-1.32 ± 0.32
2	-0.05 ± 0.13	-0.11 ± 0.12	2.03 ± 0.32	-0.85 ± 0.30
3	-0.01 ± 0.16	-0.06 ± 0.11	2.36 ± 0.29	-1.51 ± 0.37
4	-	-	0.92 ± 0.30	-1.26 ± 0.31
5	-	-	1.70 ± 0.29	-1.47 ± 0.34
**Mean ± SEM**	**-0.06 ± 0.02**	**-0.07 ± 0.01**	**1.89 ± 0.27**	**-1.28 ± 0.12**
**Mean Total ± SEM**	**-0.07 ± 0.01**		**1.59 ± 0.16**	

* The free energy difference in the unbound state should be zero as the endpoints of the transformation are chemically identical

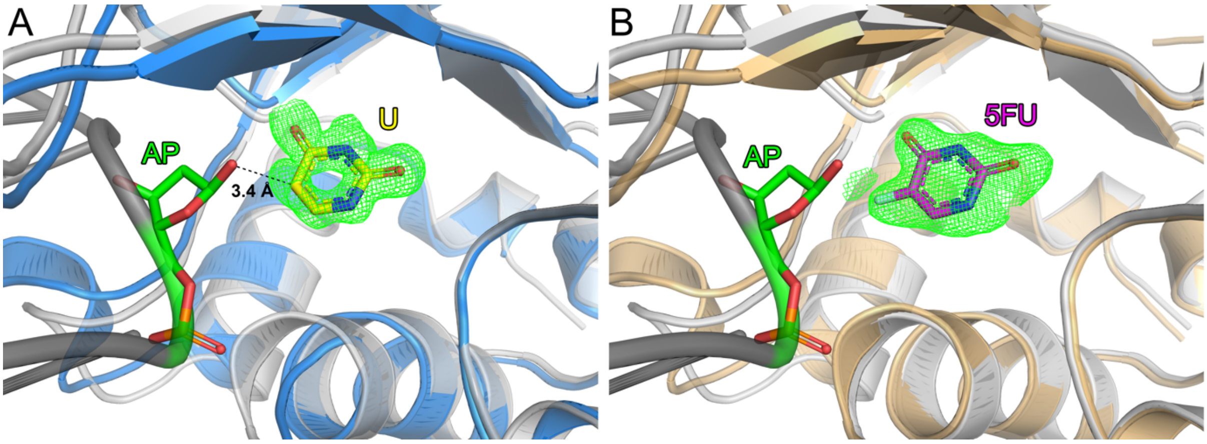

Supplementary Figure 8: Distance between AP site deoxyribose and hSMUG1 products uracil and 5FU. (A) Comparison of hSMUG1-dsDNA (light grey) with hSMUG1-Uracil (blue). The dsDNA substrate is shown as a dark grey cartoon, where the AP site is shown as a green stick representation. Uracil (U) is shown as a stick representation with C atoms coloured yellow, N atoms dark blue and O atoms red. **(B)** Comparison of hSMUG1-dsDNA (light grey) with hSMUG1-5FU (light orange). 5FU is shown as a stick representation with C atoms coloured magenta, N atoms dark blue, O atoms red and F atoms cyan. In panels **A** and **B**, a F_o-F_c omit map (green, 3.0σ) prior to ligand addition and refinement is shown. Figure produced with PyMOL (version 3.0.4, Schrödinger).

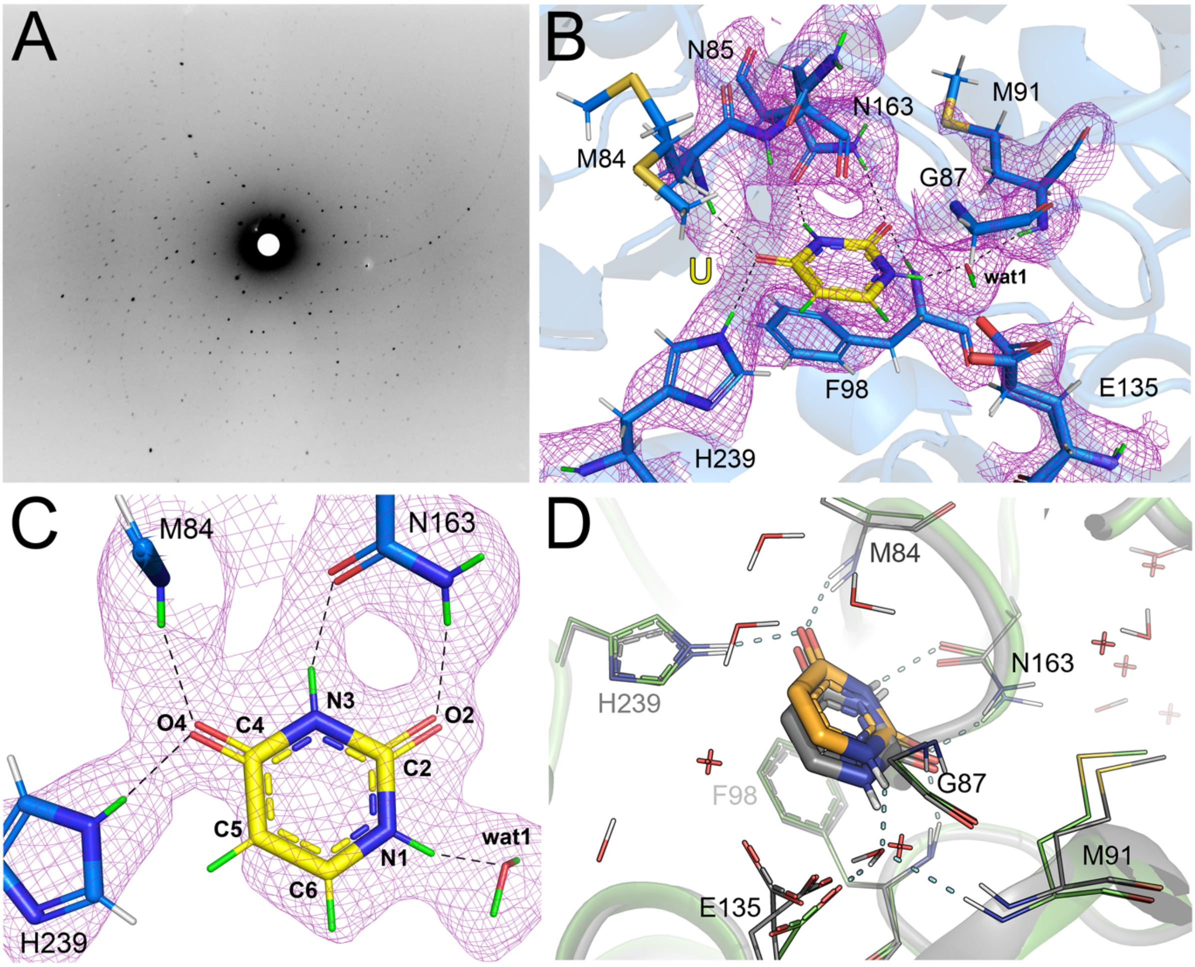

Supplementary Figure 9: hSMUG1-uracil neutron diffraction experiment. (A) hSMUG1-uracil neutron quasi-Laue diffraction pattern. **(B)** Neutron scattering density map. Apo crystals of hSMUG1 were soaked with deuterated uracil (uracil-d_2) prior to data collection. The $2F_o$-F_c neutron density in the hSMUG1 uracil binding pocket contoured at 1.0σ is depicted by purple mesh. The hSMUG1 monomer (blue) is shown as a semi-transparent cartoon. C atoms are coloured blue (hSMUG1) or yellow (uracil), O atoms red, N atoms dark blue, H atoms white and D atoms green. H-bonds are depicted as black dashed lines. **(C)** Close-up view of uracil, showing atom numbering. **(D)** Comparison of the crystal structure (dark grey) with the most representative structure from the MD simulations from the enzyme-product (hSMUG1-uracil) complex (green). Uracil is shown using sticks coloured orange (MD simulation) or grey (hSMUG1-uracil structure). Key residues are depicted as lines, and hydrogen bonds are shown as cyan dashed lines. Figures produced with PyMOL (version 3.0.4, Schrödinger).

Supplementary Table 2: Free energy differences between uracil and thymine (in kcal/mol) for each MD simulation replica in the unbound ($\Delta G_{U-T}^{unbound}$) and bound (ΔG_{U-T}^{bound}) states. Forward and backward transformations are reported separately for both the unbound and bound states alongside their respective standard errors of the mean (SEM).

Replica	$\Delta G_{\mathrm{U-T}}^{unbound}$		$\Delta G_{\mathrm{U-T}}^{bound}$	
	Forward	Backward	Forward	Backward
**1**	-6.84 ± 0.12	6.62 ± 0.12	-5.43 ± 0.37	5.05 ± 0.35
**2**	-6.79 ± 0.14	6.60 ± 0.13	-5.05 ± 0.35	4.79 ± 0.35
**3**	-6.77 ± 0.13	6.57 ± 0.12	-5.93 ± 0.42	5.70 ± 0.31
**4**	-	-	-5.50 ± 0.33	5.20 ± 0.33
**5**	-	-	-4.27 ± 0.39	4.70 ± 0.38
**Mean ± SEM**	**-6.80 ± 0.02**	**6.60 ± 0.01**	**-5.24 ± 0.24**	**5.09 ± 0.10**
**Mean Total ± SEM**	**-6.70 ± 0.04**		**-5.16 ± 0.13**	

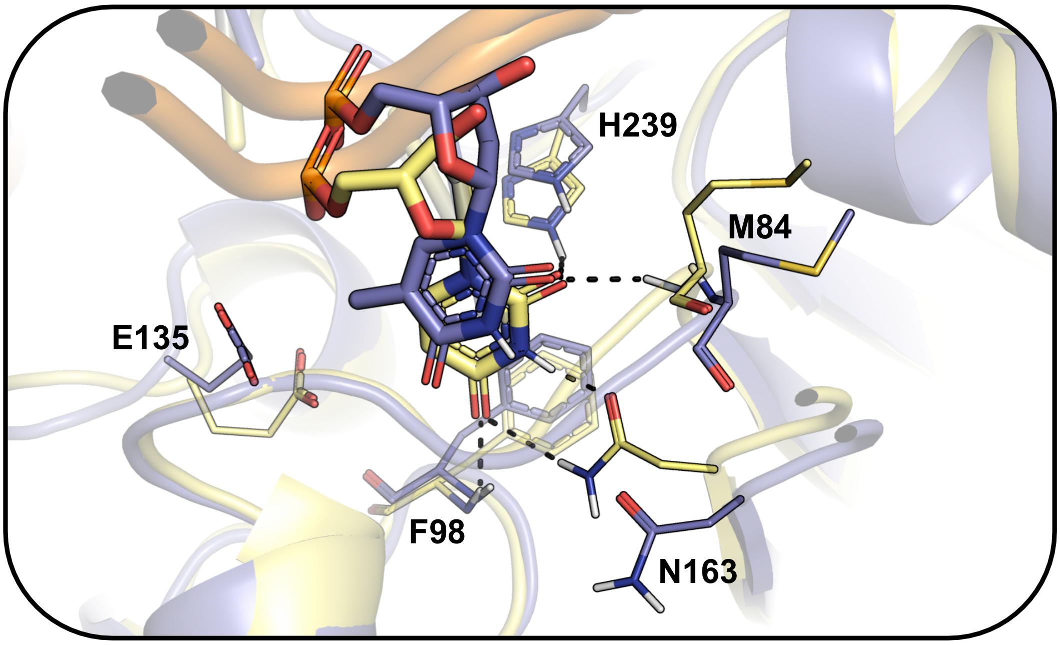

Supplementary Figure 10: Enzyme-substrate complexes representative structures. Comparison of the most representative structure from MD simulations of the binary hSMUG1-dsDNA complexes with uracil (yellow) and thymine (blue) flipped out from the dsDNA to the hSMUG1 active site. Uracil and thymine are represented as sticks and relevant residues in hSMUG1 binding site are labelled and represented as lines. Hydrogen bonds are represented as dashed black lines. Hydrogens are coloured white, oxygen red, nitrogen dark blue and sulphurs gold. DNA and phosphor atoms are coloured orange.

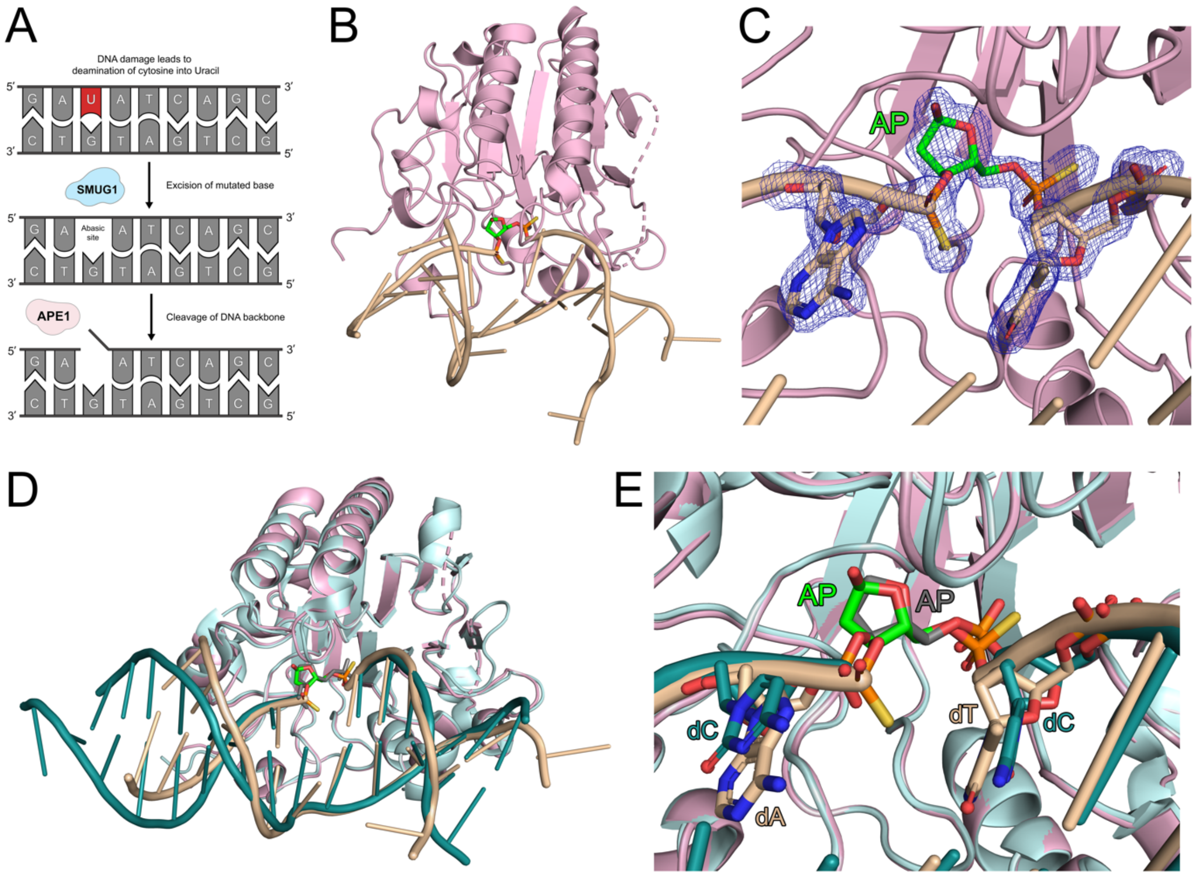

Supplementary Figure 11: hAPE1 in complex with dsDNA. (A) First steps of hSMUG1-dependent BER pathway. The repair process begins with the recognition and excision of mutagenic uracil (shown in red) from DNA by SMUG1, generating an AP site. This AP site is processed further by APE1, which creates a nick in the phosphate backbone of the AP site. **(B)** X-ray crystal structure of hAPE1 (pink) bound with dsDNA (beige). The phosphorothioated AP site in the dsDNA substrate is coloured green. **(C)** Electron density for the phosphorothioated AP site and flanking deoxynucleotides (shown as blue mesh, contoured at 1.3σ). C atoms are coloured beige, O atoms red, N atoms blue, P atoms orange and S atoms gold. **(D)** Comparison of hAPE1-dsDNA structures. Cartoon monomers are coloured light pink (APE1 structure from current work) or light cyan (PDB ID: 1DEW). The dsDNA products are coloured either beige with AP site shown in green (current work) or dark teal with AP site coloured dark grey (1DEW). RMSD following Cα-atom superposition =0.39 Å. **(E)** Zoomed in view of panel **D**, showing the AP site. Deoxyadenosine (dA), deoxycytosine (dC) and deoxythymidine (dT) are shown as sticks. Figure produced with PyMOL (version 3.0.4, Schrödinger).

Supplementary Table 3. Oligonucleotides used in this study.

Oligo #	Vendor	Nucleotide sequence	Description
1	GenScript	5′-CGGACTCACGGG-3′	Unmodified, sense strand
2	GenScript	5′-CGGACTUACGGG-3′	Uracil-containing DNA, sense strand
3	GenScript	5′-CGGACT/idSp/ACGGG-3′	AP site modification, sense strand
4	GenScript	5′-CGGACT*U*ACGGG-3′	Phosphorothioated around uracil base, sense strand
5	GenScript	5’-CCCGTGAGTCCG-3’	Unmodified, anti-sense strand
6	GenScript	5′-CCCGTGAGTCCG-3′Cy5	Unmodified, anti-sense strand, Cy5-labelled
7	GenScript	5′-CGGACTUACGGG-3′Cy5	Uracil-containing DNA, sense strand, Cy5-labelled

Supplementary Table 4. Relevant dsDNA and ssDNA oligos used for crystallographic and MST experiments. See **Supplementary Table 3** for oligonucleotide sequence information.

**Oligo name**	**Details**	**Description**
Crystallography		
dsDNA-uracil	Oligo 2 + Oligo 5 (1:1 ratio)	Uracil-containing dsDNA
dsDNA-uracil*	Oligo 4 + Oligo 5 (1:1 ratio)	dsDNA phosphothioated around uracil nucleotide
MST experiments		
dsDNA-Cy5	Oligo 1 + Oligo 6 (1:1 ratio)	Unmodified dsDNA, labelled with Cy5
dsDNA-uracil-Cy5	Oligo 2 + Oligo 6 (1:1 ratio)	Uracil-containing dsDNA, labelled with Cy5
dsDNA-AP-Cy5	Oligo 3 + Oligo 6 (1:1 ratio)	dsDNA with AP site modification, labelled with Cy5
ssDNA-Cy5	Oligo 6 only	Unmodified ssDNA, labelled with Cy5
ssDNA-uracil-Cy5	Oligo 7 only	Uracil-containing ssDNA, labelled with Cy5

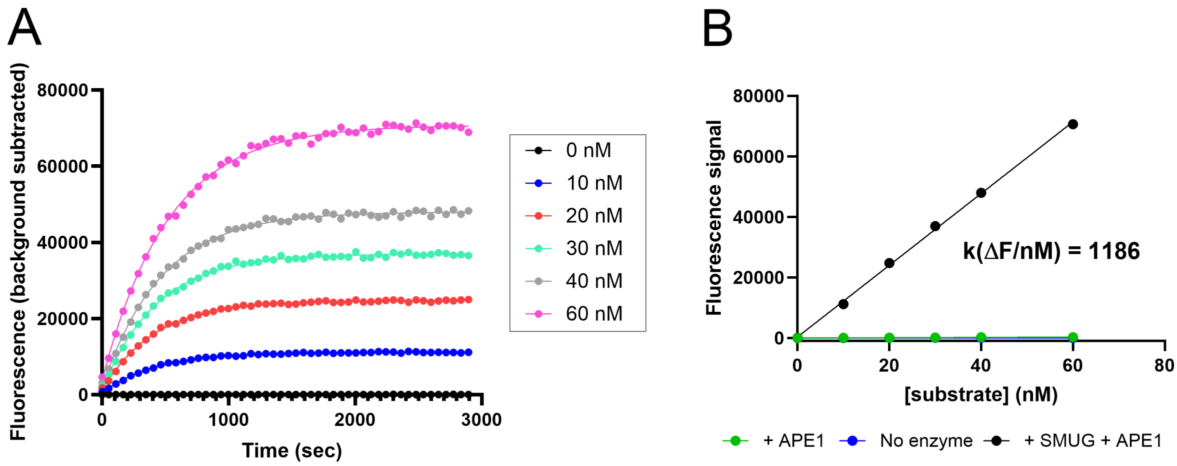

Supplementary Figure 12: hSMUG1 activity measurements and controls. (A) Time course of conversion of different concentrations of SMUG1 substrate to product was measured as FAM fluorescence (Exc485/Em535). An excess of hSMUG1 (10 nM) and APE1 (10 nM) was added and fluorescence was followed over time. **(B)** A standard curve was created by plotting the maximal fluorescence values from **A** at the different substrate concentrations, which represent full conversion to product, against substrate concentration. Controls lacking both SMUG1 and APE1 or lacking SMUG1 only were included to show that fluorescence signal is dependent on SMUG1 only. Linear regression was performed using GraphPad Prism 8.0. Experiments were performed twice with data points in triplicate. Data are presented as mean ± SD (n=2).

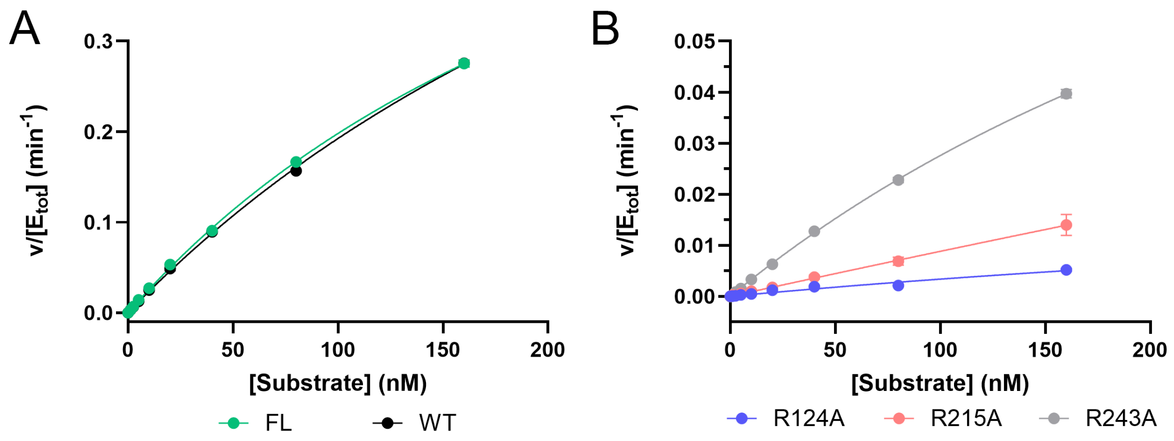

Supplementary Figure 13: Fluorescence-based hSMUG1 activity assay saturation curves. (A) hSMUG1 wt variants. FL: Full-length hSMUG1. WT: N-terminally truncated hSMUG1-Δ24, used as "wild-type" protein for enzyme assays, crystallography and MST studies. **(B)** Saturation curves of hSMUG1 R124A, R215A and R243A mutants. Experiments were performed twice with data points in triplicate. Data are presented as mean ± SD (n=2).

Supplementary Table 5. Kinetic parameters of SMUG1 variants.

**hSMUG1 variant**	k_{cat} (min^{-1})	K_m (nM)	k_{cat}/K_m ($M^{-1}min^{-1}$)
FL WT	0.80 ± 0.03	294 ± 12	$2.6 \times 10^6 \pm 3.5 \times 10^3$
WT Δ24	0.95 ± 0.02	393 ± 10	$2.4 \times 10^6 \pm 2.0 \times 10^4$
R124A	-*	-*	$4.0 \times 10^4 \pm 9.5 \times 10^3$
R215A	-*	-*	$9.6 \times 10^4 \pm 5.6 \times 10^2$
R243A	0.15 ± 0.01	432 ± 28	$3.4 \times 10^5 \pm 3.9 \times 10^3$

Parameters were determined from two biological replicates using initial rates calculated using linear regression and data points in triplicate at each substrate concentration. The Michaelis Menten equation was fit to the data points using GraphPad Prism 8.0. k_{cat}/K_m values were determined by fitting the equation $v = k_{cat}/K_m \times ([S]/(1+([S]/Km)))$ to the data. *Poor substrate saturation made it impossible to determine k_{cat} and K_m-values. Experiments were performed twice with data points in triplicate. Data are presented as mean ± SD (n=2).

Supplementary Table 6. Dissociation constants (Kd) for wild-type hSMUG1 and hSMUG1-N85A variants binding to single- and double-stranded DNA substrates measured via MST.

Oligonucleotide	WT<br>Kd (nM)	N85A<br>Kd (nM)
**No modification, dsDNA**<br>(5′-CGGACTCACGGG-3′)	1267 ± 49	1043 ± 26
**dU, dsDNA**<br>(5′-CGGACTUACGGG-3′)	7 ± 0.5	7 ± 0.3
**No modification, ssDNA**<br>(5′-CCCGTGAGTCCG -3′Cy5)	1305 ± 56	1087 ± 61
**dU, ssDNA**<br>(5′-CGGACTUACGGG -3′Cy5)	1081 ± 122	595 ± 33

NB, no detectable binding under the conditions tested. WT denotes the N-terminally truncated hSMUG1-Δ24 construct, which was used throughout this study as the wild-type protein for crystallography, enzymatic assays, and MST measurements. Kd and standard deviations (mean values ± SD) were calculated using fits from at least three individual titrations.

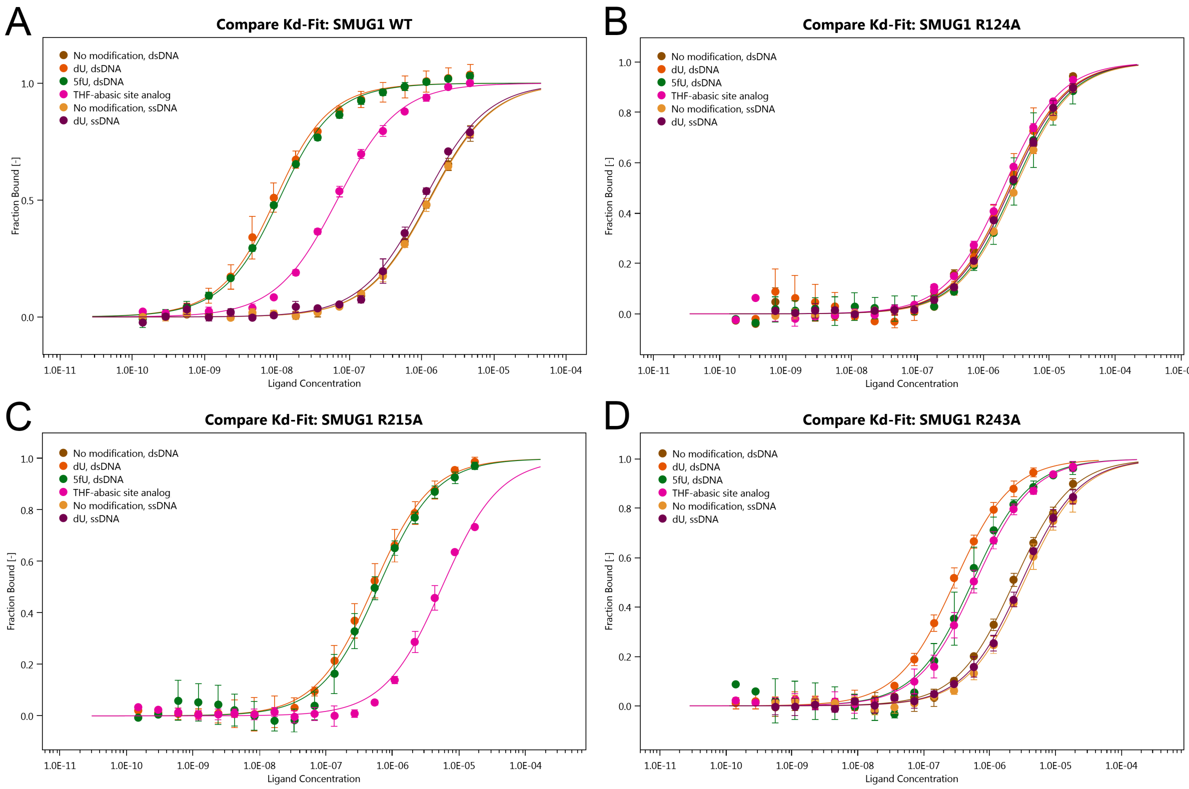

Supplementary Figure 14: Binding curves of hSMUG1 variants to DNA substrates measured by MST. (A) Wild-type hSMUG1, **(B)** hSMUG1-R124A, **(C)** hSMUG1-215A, and **(D)** hSMUG1-243A. Brown circles indicate unmodified dsDNA; orange circles, uracil-containing dsDNA; green circles, 5FU–containing dsDNA; magenta circles, dsDNA containing a tetrahydrofuran AP-site analogue; light brown circles, unmodified ssDNA; and purple circles, uracil-containing ssDNA. Lines represent fits of the data points using the K_D fit derived from the law of mass action. K_D and standard deviations (mean ± SD) were calculated by the analysis software using fits from three individual titrations.

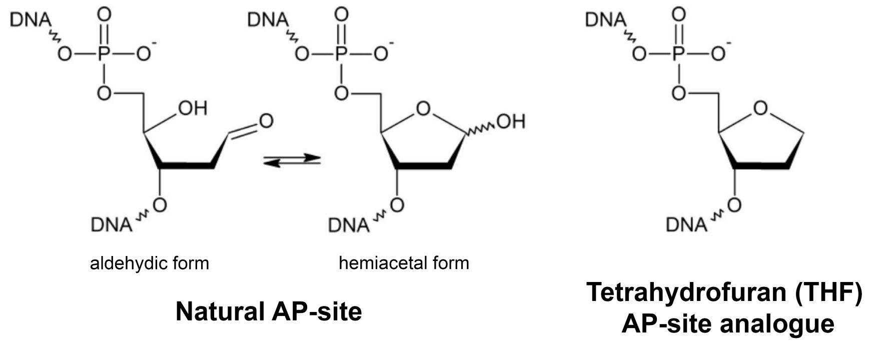

Supplementary Figure 15. Structures of the naturally occurring AP-site and a synthetic tetrahydrofuran (THF) AP-site analogue. In the current study, MST measurements were performed using a dsDNA substrate containing the tetrahydrofuran analogue, which is chemically distinct from the naturally occurring AP site.

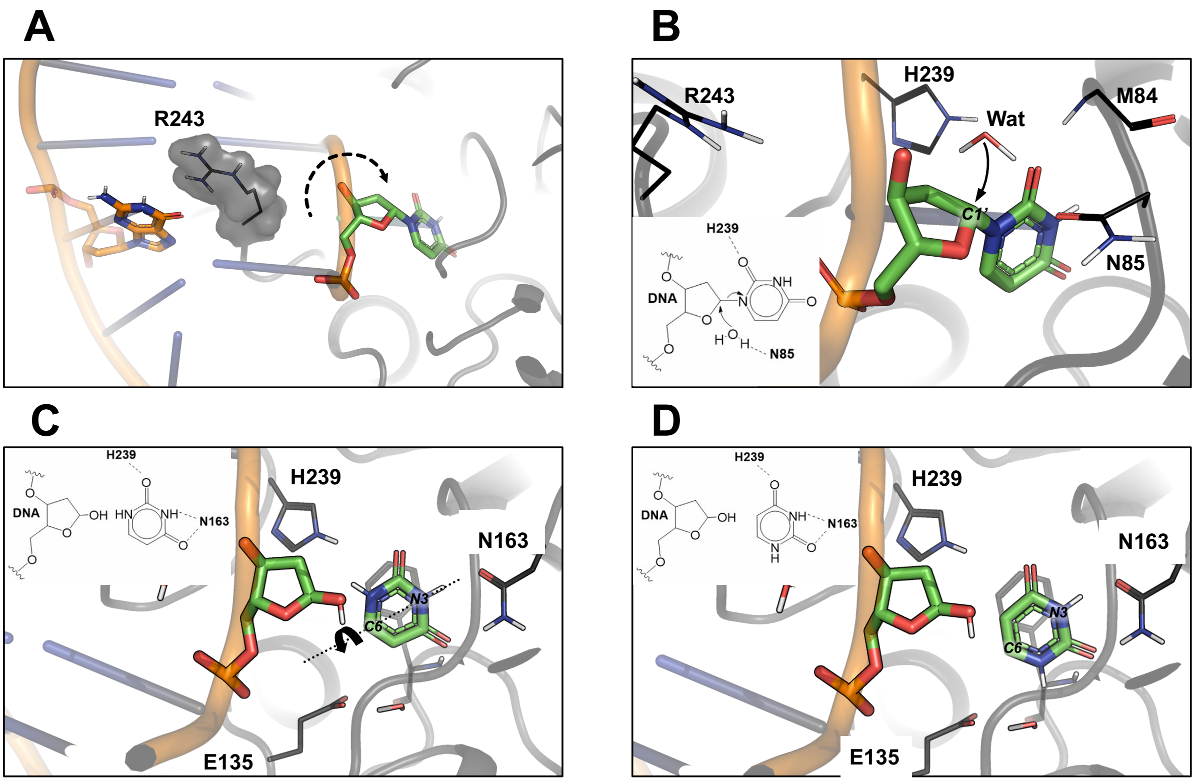

Supplementary Figure 16. Proposed hSMUG1 mechanism of action. (A) Overview of uracil recognition and base flipping. R243 (dark grey surface) inserts into the DNA duplex, flipping out the base into the hSMUG1 active site (discontinuous curve line). **(B)** Active site stabilization and catalysis. M84 and H239 stabilize the flipped-out substrate through interactions with the carbonyl oxygen, while N85 coordinates a catalytic water molecule. Nucleophilic attack of this water (Wat) on the C1' atom of the deoxyribose (black arrow) leads to cleavage of the N-glycosidic bond, releasing uracil and generating an abasic AP site. **(C-D)** hSMUG1-dsDNA-uracil product ternary complex. Following cleavage, the uracil can adopt either the native or rotated binding pose within the binding site. The rotated pose may prevent reformation of the glycosidic bond and disfavour the backward reaction. 2D representations are shown in **B**, **C** and **D**. The uracil nucleotide and product are shown as green sticks, relevant hSMUG1 residues are labelled and shown as black lines. DNA and phosphor atoms are coloured orange, oxygen red, nitrogen dark blue, carbon green and hydrogen white.

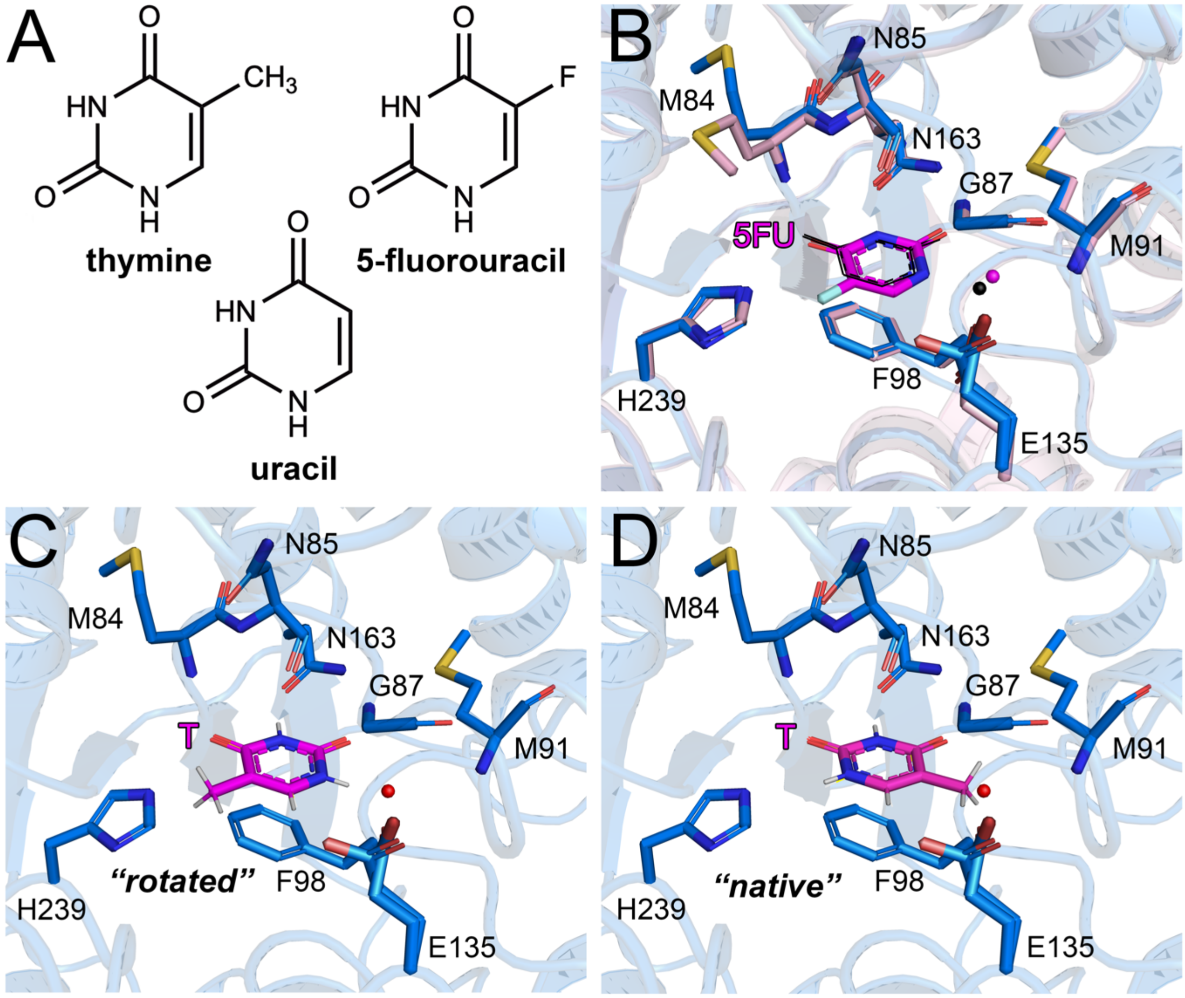

Supplementary Figure 17: Structural insights into hSMUG1 uracil specificity. (A) Chemical structure of uracil, 5-flururacil (5FU) and thymine. **(B)** Comparison of hSMUG1-uracil (blue) with hSMUG1-5FU (light pink) where protein monomers are displayed as cartoon representations. Uracil (U) is shown as thick black lines. 5FU is shown as sticks. C atoms are coloured magenta, O atoms red, N atoms dark blue and F atoms cyan. Water molecules are shown as black (uracil complex) or magenta (5FU complex) spheres. **(C)** hSMUG1-uracil binding pocket where thymine is superimposed on uracil in the “rotated” conformation. **(D)** hSMUG1-uracil binding pocket where thymine is superimposed on uracil in the “native” conformation. Figure produced with PyMOL (version 3.0.4, Schrödinger).

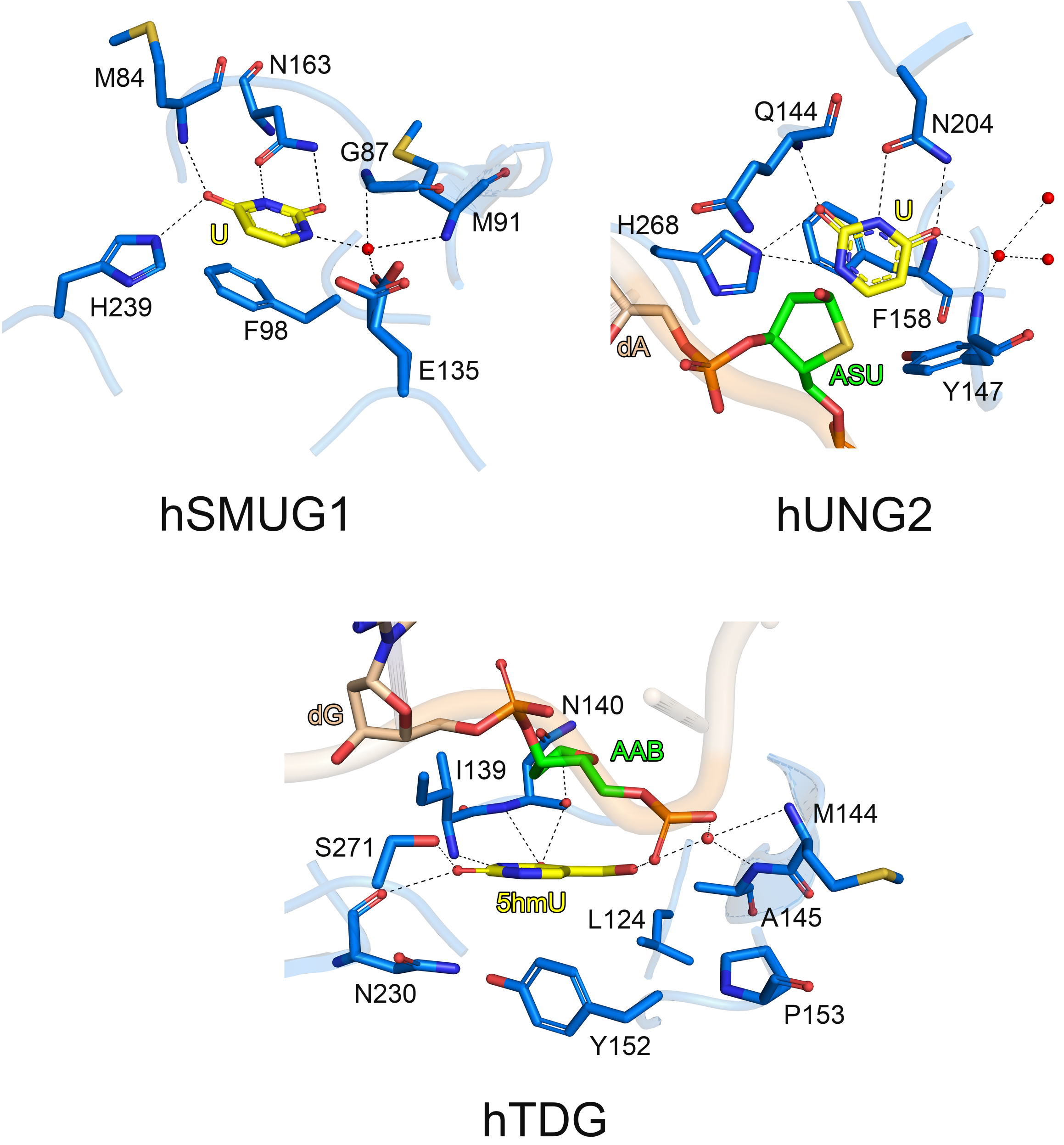

Supplementary Figure 18: Comparison of uracil binding pockets of hSMUG1 and other members of the UDG protein superfamily. The H-bond networks for the uracil binding pocket of hSMUG1-uracil (current work), hUNG2-dsDNA-uracil (PDB ID: 1EMJ) and hTDG-dsDNA-5hmU (PDB ID: 4FNC) are shown, where hydrogen bonds are depicted as dashed lines. Amino acids contributing to ligand coordination are shown as sticks, where C atoms are coloured blue, O atoms red, N atoms dark blue and S atoms gold. Uracil (U) and 5hmU are shown as sticks, C atoms coloured yellow. Deoxyguanosine (dG) and deoxyadenosine (dA), deoxycytosine (dC) are shown as sticks, C atoms coloured beige and P atoms coloured orange. The modified AP-sites (ASU: 4'-thio-2'4'-dideoxyribofuranose-5'-phosphate and AAB: 2'-deoxy-ribofuranose-5'-monophosphate) for the dsDNA substrates in the hUNG2 and hTDG structures are shown as sticks, C atoms coloured green. Water molecules are shown as red spheres. Figure produced with PyMOL (version 3.0.4, Schrödinger).

Supplementary Table 7. Data collection and refinement statistics for hSMUG1 and hAPE1 crystal structures.

Complex identifier	hSMUG1-apo	hSMUG1-uracil	hSMUG1-dsDNA	hAPE1-DNA	hSMUG1-5FU
**Data collection**					
PDB code	9GGS	9GK0	9GM2	9RQS	9RQP
Station	DLS-I04	MAXIV-BioMAX	DLS-I24	DLS-I03	DLS-I04
Space group	$P2_12_12_1$	$P2_12_12_1$	C2	C2	$P2_12_12_1$
Cell dimensions:					
a, b, c (Å)	49.1, 60.6, 91.9	49.1, 60.7, 92.0	150.1, 84.4, 88.3	70.12, 71.71, 75.27	48.56, 59.98, 91.29
α, β, γ (°)	90.0, 90.0, 90.0	90.0, 90.0, 90.0	90.0, 113.4, 90.0	90.0, 110.57, 90.0	90.0, 90.0, 90.0
Resolution (Å)	50.6-1.38 (1.40-1.38)	27.4-0.95 (0.97-0.95)	71.9-2.12 (2.16-2.12)	38.9-1.73 (1.73-1.70)	50.1-1.90 (1.93-1.90)
Total reflections	687394 (17790)	4556742 (219417)	721669 (36541)	261120 (13298)	267749 (9878)
Unique reflections	56140 (2278)	172475 (8380)	56982 (2865)	38179 (2031)	21518 (1090)
R_{merge}	0.074 (0.767)	0.113 (3.378)	0.402 (5.327)	0.066 (0.989)	0.280 (3.498)
R_{pim}	0.031 (0.402)	0.031 (0.941)	0.117 (1.553)	0.041 (0.636)	0.083 (1.244)
$CC_{1/2}$	0.999 (0.764)	0.998 (0.442)	0.994 (0.328)	0.999 (0.876)	0.993 (0.708)
I/σ	16.1 (2.1)	12.4 (1.1)	5.9 (0.8)	12.7 (1.3)	7.5 (0.8)
Completeness	98.2 (80.9)	99.8 (99.1)	100 (100)	99.3 (99.0)	99.2 (100)
Redundancy	12.2 (7.8)	26.4 (26.2)	12.7 (12.8)	6.8 (6.5)	12.4 (9.1)
**Refinement**					
R_{work}/R_{free} (%)	15.2/17.3	13.6/14.6	20.6/25.3	19.3/22.8	19.0/23.1
B-factors:					
Protein	16.7	14.5	28.4/28.5/33.2/32.4	16.7	28.1
Ligand of interest	34.5	24.9	59.6/78.9/59.6/75.7	44.4/37.5	47.8
Water	29.7	37.1	35.5	34.7	33.0
R.m.s. deviations:					
Bond lengths (Å)	0.013	0.005	0.005	0.007	0.007
Bond angles (°)	2.10	0.85	1.42	1.77	1.65
Ramachandran plot:					
Favoured (%)	96.72	97.52	97.04	97.71	96.72
Allowed (%)	2.46	2.07	2.55	2.29	3.28
Outliers (%)	0.82	0.41	0.41	0.0	0.0

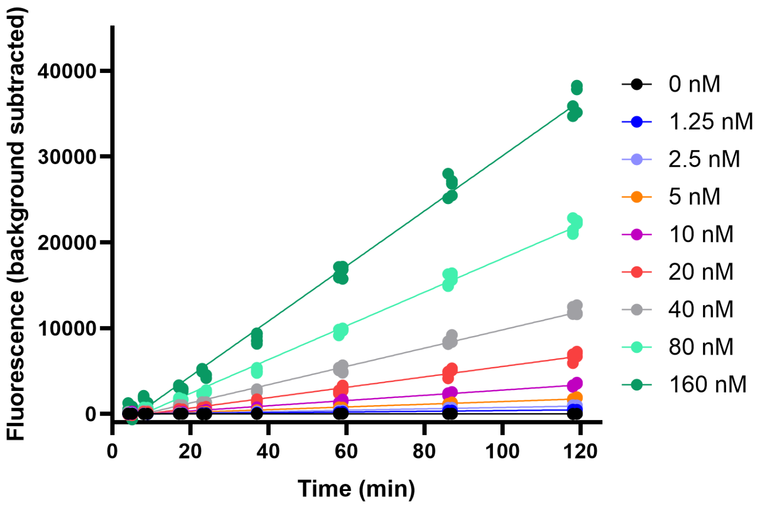

Supplementary Figure 19: **Increase in FAM fluorescence with time, representing SMUG1 catalyzed formation of product.** Initial rates of hSMUG1 at different SMUG1 substrate concentrations were determined using linear regression (GraphPad Prism 8.0) and were subsequently used to generate saturation curves and to determine kinetic parameters. Graph shows two independent experiments with data points performed in triplicate (n=2).

Supplementary Table 8. Data collection and refinement statistics of X-ray and neutron diffraction experiment for hSMUG1-deuterated uracil complex (joint X/N refinement).

Dataset	X-ray	Neutron
**Data collection**		
PDB code	9SQ2	
Station	ESRF-BM07-FIP2	ILL-LADI-DALI
Space group	$P2_12_12_1$	
Cell dimensions:		
a, *b*, *c* (Å)	49.9, 61.1, 93.9	
α, *β*, *γ* (°)	90.0, 90.0, 90.0	
Resolution (Å)	46.93-1.54 (1.60-1.54)	30.36-2.30 (2.42-2.30)
Total reflections	548305 (51355)	78662 (8290)
Unique reflections	42729 (3969)	11690 (1523)
R_{merge}	0.088 (1.958)	0.23 (0.497)
R_{pim}	0.026 (0.578)	0.087 (0.193)
$CC_{1/2}$	0.999 (0.486)	0.994 (0.591)
I/σ	17.8 (1.4)	8.7 (2.2)
Completeness	99.5 (95.8)	90.6 (83.7)
Redundancy	12.8 (12.9)	6.7 (5.4)
**Refinement**		
R_{work}/R_{free} (%)	15.75/17.92	23.95/27.50
B-factors:		
Protein	30.79	
Ligand of interest	40.08	
Water	42.56	
R.m.s. deviations:		
Bond lengths (Å)	0.016	
Bond angles (°)	1.37	
Ramachandran plot:		
Favoured (%)	97.54	
Allowed (%)	2.05	
Outliers (%)	0.41	
